# Inhibiting YAP1 reduced abdominal aortic aneurysm formation by suppressing adventitial fibroblast phenotype transformation and migration

**DOI:** 10.1111/jcmm.70159

**Published:** 2024-11-04

**Authors:** Cuiping Xie, Yanting Hu, Zhehui Yin

**Affiliations:** ^1^ Department of General Intensive Care Unit, Key Laboratory of Early Warning and Intervention of Multiple Organ Failure, Second Affiliated Hospital Zhejiang University School of Medicine Hangzhou China; ^2^ Department of Cardiology, Cardiovascular Key Laboratory of Zhejiang Province, Second Affiliated Hospital Zhejiang University School of Medicine Hangzhou China

**Keywords:** abdominal aortic aneurysm, adventitial fibroblasts, myofibroblasts, YAP1

## Abstract

The adventitial fibroblast (AF) is the most abundant cell in the vascular adventitia, a few studies had confirmed that AF contributed to abdominal aortic aneurysm (AAA) development; YAP1 involved in several vascular diseases by promoting AF transformed to myofibroblast, the role of YAP1 in AAA is not clear yet. This study aims to determine whether YAP1 play a role in AAA process by regulating AF function. We found the expression of YAP1was significantly increased in aneurysm tissues of AAA patients compared to normal adjacent vascular tissues and mainly in adventitia. YAP1 also upregulated in elastase‐induced and CaCl_2_‐induced mice AAA model. Suppressed YAP1 function with YAP1 inhibitor‐Verteporfin declined AAA incident rate remarkably in mice, and the collagen deposition in the adventitia was alleviated obviously. Afterwards, we studied the effect of YAP1 on the function of AF, Verteporfin was used to block YAP1 in vitro, the process of AF transforming to myofibroblast and migration were almost completely eliminated after inhibiting YAP1 expression. This study demonstrated that YAP1 may play a key role in AAA development, inhibiting YAP1 significantly reduced AAA formation through suppressed the process of AF transformed to myofibroblast and migration.

## 1 INTRODUCTION

Abdominal aortic aneurysm (AAA) is a chronic inflammatory disease characterized by permanent expansion of local abdominal aortic segments, which is more common in elderly men over 65 years old. Aneurysm rupture is the most serious complication, the mortality rate of which is more than 80%. It is a high‐risk vascular disease that leads to an estimated 160,000 deaths worldwide each year.^1^

Previous studies on AAA mainly focused on apoptosis and inflammation of vascular smooth muscle cells, while adventitial fibroblasts (AF), the most abundant cell in the outer membrane of blood vessels, have been ignored. As a receptor of vascular environmental pressure, AF responds to stimulation in a variety of ways, including proliferation, secretion of cytokines, chemokines and growth factors, regulation of extracellular matrix, dilation of nourish blood vessels. AF can exhibit remarkable plasticity, acquire a smooth muscle cell phenotype (myofibroblast), and participate in vascular remodelling or acquire a pro‐inflammatory phenotype through epigenetic changes, thereby recruiting monocytes and macrophages.^2^, ^3^

Studies have shown that myofibroblasts of the adventitia are involved in the pathological processes of AAA, atherosclerosis, vascular stenosis, pulmonary hypertension and graft vein remodelling.^4^, ^5^, ^6^ Whether AF phenotypic transformation plays a protective or promoting role in AAA is still need to be confirmed, and the specific mechanism involved has not been fully elucidated.

YAP1 plays an important role in vascular remodelling,^7^ YAP1 can regulate the function of AF through various ways; changes in mechanical stress and hemodynamics can activate YAP1, and subsequently YAP1 regulates the phenotypic transformation and migration of AF, thereby causing vascular matrix remodelling.^8^, ^9^, ^10^ The phenotypic transformation of AF is one of the important pathological mechanisms of AAA. Whether YAP1 is involved in this process and ultimately affects the occurrence and development of AAA is still unclear.

## 2 MATERIALS AND METHODS

### 2.1 Human sample and ethics

This study was approved by the Ethics Committee of the Second Affiliated Hospital of Zhejiang University School of Medicine (approval no. AIRB‐2022‐06016). All subjects provided informed consent prior to their inclusion in the study, and the experiments complied with the principles outlined in the Declaration of Helsinki. Human AAA tissues were obtained from AAA patients during the operation, the tissues were fixed with 4% formalin and then embedded with paraffin to prepare tissue microarray sections for immunohistochemical experiments, or stored in liquid nitrogen for subsequent molecular experiments.

### 2.2 Mice

All animal study protocols were approved by the Animal Care and Use Committee of the Second Affiliated Hospital of Zhejiang University School of Medicine (approval no. IRB‐2022‐0400). All animal studies were performed at the Key Laboratory of Early Warning and Intervention of Multiple Organ Failure and Cardiovascular Key Laboratory of Zhejiang Province, Second Affiliated Hospital. Eight to ten weeks male C57BL/6J mice were purchased from Shanghai SLAC Laboratory Animal Company. Mice were bred in house under specific pathogen‐free conditions with free access to a normal chow diet and water, at a constant temperature (22 ± 2°C) and humidity (60%–65%) with a 12 h dark/light cycle.

### 2.3 Construction of mice abdominal aortic aneurysm model

Elastase‐induced and CaCl_2_‐induced mice AAA model were used in this study as described previously.^11^ Mice were anaesthetized with 2%–3% isoflurane, and the infrarenal abdominal aorta was isolated completely, then enfolded with a small piece of gauze soaked in elastase (2.5 U/mL, Cat#E1250, Sigma‐Aldrich) or CaCl_2_ (0.5 M, Cat#C5670, Sigma‐Aldrich) for 15 min, then intra‐abdominal was washed with saline, mice were executed under deep isoflurane anaesthesia followed by two cervical dislocation at the 14 day, the aortas (from the aortic root to the iliac bifurcation) were isolated for embedding or the measurement of aortic diameter or subsequent molecular experiments. To determine the effect of suppressing YAP1 on AAA, mice was pretreated with YAP1 inhibitor‐Verteporfin (Cat#HY‐B0146, MCE; 30 μg/g per time, the Verteporfin working solution configuration method: add each solvent in sequence: 10% DMSO→40% PEG300→5% Tween‐80→45% Saline, the final concentration is 5 mg/mL) for 24 h before the operation in both AAA models, then followed by same research processes mentioned above.

### 2.4 Quantification of abdominal aortic dilation

Incidence of AAA was defined by either (1) 50% or more increase of the maximal diameter in the infrarenal aortic region as compared to the baseline or (2) death due to abdominal aortic rupture.^12^ At termination, aortas were dissected and placed in 10% neutrally buffered formalin. Maximal diameter of infrarenal aortas was measured ex vivo as a parameter for AAA quantification using Image J software. All the data were quantified by two observers that were blinded to the study design.

### 2.5 Isolation and culture of primary AF

Eight‐week‐old male C57BL/6J mice were selected and executed by deep isoflurane anaesthesia followed by two cervical dislocation. Abdominal aortas were separated and cut open, the media and intima were striked off, then the adventitial membrane was chopped to the size of 1–2 mm^2^ and digested by type II collagenase (Cat#17101015, Thermo Fisher Scientific), cells were cultured and passed when the length reached 80%–90%, 3–5 passages were used for follow‐up experiments. The purity of primary adventitial fibroblasts was verified by positive immunofluorescent staining of vimentin and negative staining for α‐SMA.^11^

### 2.6 Wound healing

The AF was plated into the 6‐well plate and grew to 100% density, and the 200 μL pipette tip was vertically placed at the bottom of the plate to make linear scratch, then washed twice with PBS, and pretreated with Verteporfin (1 μM) for 24 h, and then recombinant mouse TGF‐β1 (10 ng/mL, Cat#C16W, Novoprotein) was added to continue incubation for 24 h. Take pictures at 0, 12 and 24 h. The cell migration rate is calculated as (A0‐AN)/A0 × 100, where A0 represents the initial scratch distance and AN represents the scratch distance at the shooting time point.

### 2.7 AF phenotypic transformation

AF was plated into 6‐well plates, and after being pretreated with (1 μM) for 24 h, cells were obtained after incubation with TGF‐β1 (10 ng/mL) for 24 h. The expression of AF to myofibroblast transformation marker‐α‐SMA was observed by western blotting.

### 2.8 Western blotting

Protein lysate samples were prepared from snap frozen aortas and cells in RIPA solution (Cat# P0013B; Beyotime) supplemented with protease inhibitor (Cat# 05892791001; Roche). Denatured protein lysates were resolved by 8%–10% (wt/vol) SDS‐PAGE gels. After transfer, membranes were blocked in 5% (wt/vol) non‐fat dry milk diluted in PBS and incubated with primary antibodies against YAP1 (14,074, CST, 1:1000), α‐SMA (ab124964, abcam, 1:1000), β‐actin (ab197345, abcam, 1:1000) or β‐tubulin (ab6046, abcam, 1:1000) overnight at 4°C and subsequently incubated with horseradish peroxidase conjugated secondary antibodies which were detected by enhanced chemiluminescence (Cat# WBKLS0500; Millipore). Immunoblots were analysed using ImageLab software (Bio‐Rad).

### 2.9 Statistical analysis

GraphPad Prism 9 was used for statistical analyses. To compare continuous response variables between two groups, an unpaired two‐tailed Student's *t*‐test was applied for normally distributed variables that passed the equal variance test, and Mann–Whitney *U* test was used for variables not passing either normality or equal variance test. To compare more than two groups, one‐way ANOVA and Holm–Sidak method was performed for normally distributed variables that passed equal variance test and Kruskal–Wallis one way ANOVA on Ranks with Dunn method for variables failed to pass normality or equal variance test, respectively. *p* < 0.05 was considered as statistically significant.

## 3 RESULTS

### 3.1 YAP1 was increased in the human AAA tissue

**FIGURE 1:**
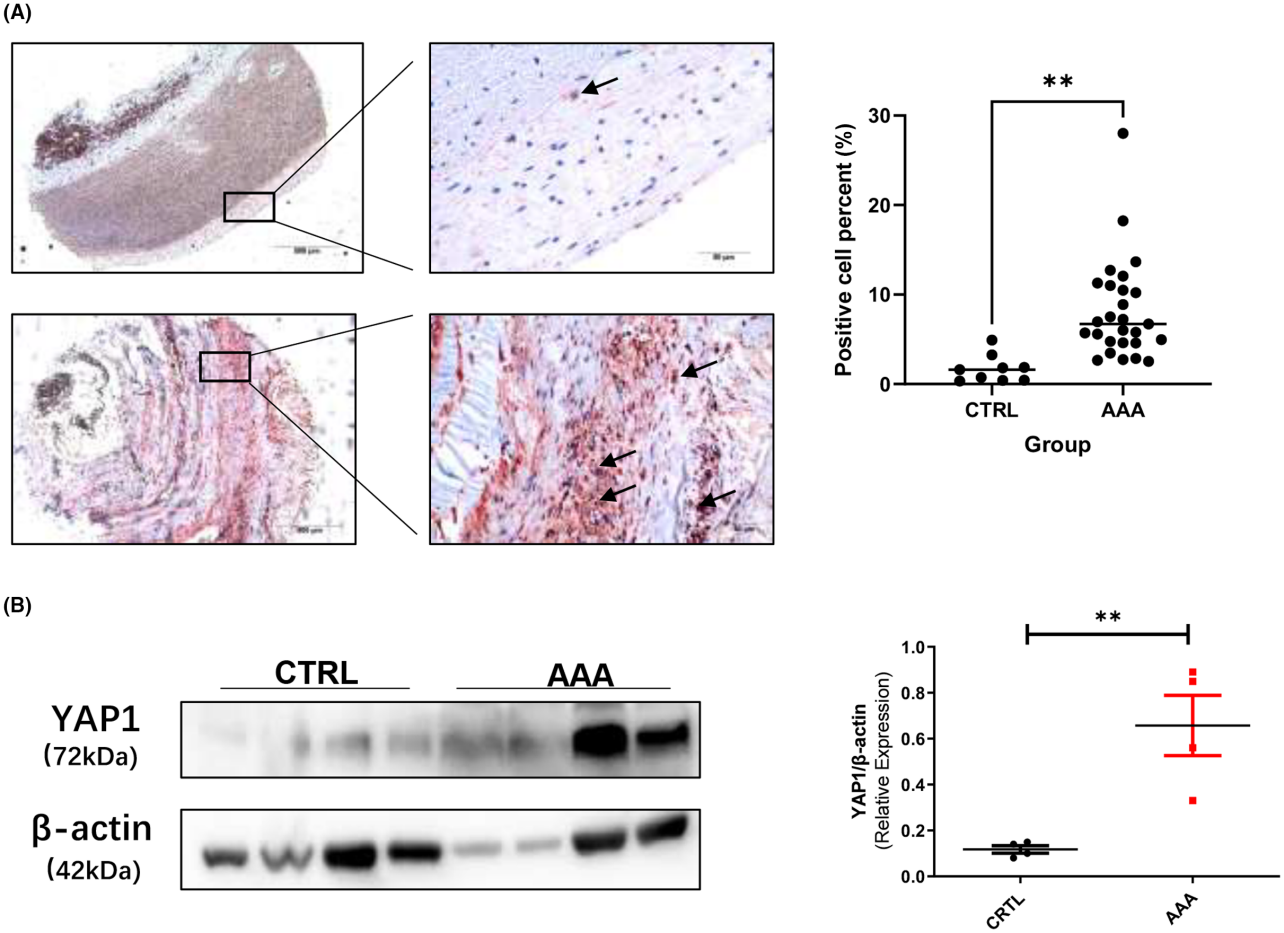
YAP1 was increased in the human AAA adventitial tissue. (A) Representative images of YAP1 expression in aneurysmal lesions (*n* = 26) and normal aorta lesions adjacent to the aneurysm (*n* = 9) as revealed by YAP1 immunostaining using tissue microarray sections; Black boxes indicate the aorta adventitia, black arrows indicate YAP1 positive location; Scale bars represent 500 μm (left images) and 50 μm (right images), YAP1 positive cell numbers were counted and analysed with Student *t‐*test; (B) Protein level of YAP1 in normal aorta lesions adjacent to the aneurysm (*n* = 4) and aneurysmal lesions (*n* = 4), Student *t‐*test was used for data analysis. Values were represented as mean ± SEM; **p < 0.01.

We performed immunohistochemical staining (IHC) using tissue microarray sections confirmed that YAP1 was significantly increased in AAA tissues, and was mainly expressed in the adventitial membrane (Figure 1A). Meanwhile, we compared human AAA tissues to normal blood vessel tissues near aneurysms with western blotting test also showed that YAP1 expression was increased in AAA (Figure 1B).

### 3.2 YAP1 was increased in mice AAA tissues

**FIGURE 2:**
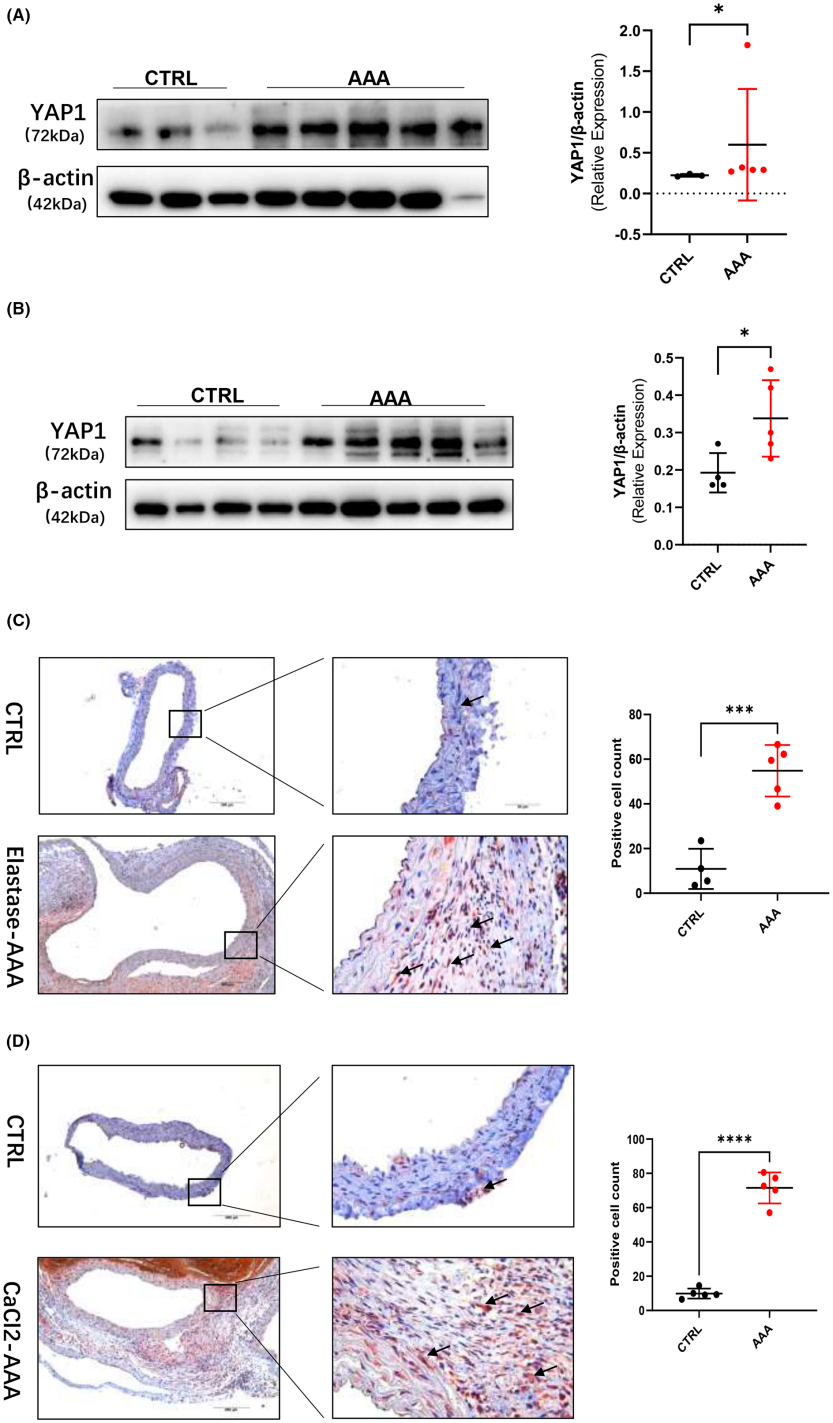
YAP1 was increased in mice AAA tissues. (A) Protein level of YAP1 in control aortas (*n* = 3) and elastase‐induced aneurysmal lesions (*n* = 5); (B) Protein level of YAP1 in control aortas (*n* = 4) and CaCl2‐induced aneurysmal lesions (*n* = 5); (C) Representative images of YAP1 expression in control aortas (*n* = 4) and elastase‐induced aneurysmal lesions (*n* = 5) as revealed by YAP1 immunostaining; (D) Representative images of YAP1 expression in control aortas (*n* = 5) and CaCl_2_‐induced aneurysmal lesions (*n* = 5) as revealed by YAP1 immunostaining scale bars represent 200 μm (left images) and 50 μm (right images). Black arrows indicate YAP1 positive location. Student *t‐*test was used for data analysis in (A–D). Values were represented as mean ± SEM; *p < 0.05; ***p < 0.001, ****p < 0.0001, respectively.

After the successful establishment of elastase‐induced AAA model and CaCl_2_‐induced AAA model, the expression of YAP1 in the control abdominal aorta and AAA tissues was also detected by western blotting and immunohistochemical technique. The expression of YAP1 was increased in the elastase‐induced AAA (*n* = 5) tissue compared with that in the control group (saline, *n* = 3) (Figure 2A); likewise, the expression of YAP1 in the CaCl_2_‐induced AAA (*n* = 5) tissue was significantly increased compare to the control group (saline, *n* = 4) (Figure 2B). IHC also confirmed that YAP1 expression was increased in AAA tissues of both models, especially in the adventitial membrane (Figure 2C,D).

### 3.3 AF phenotypic transformation involved in AAA process

**FIGURE 3:**
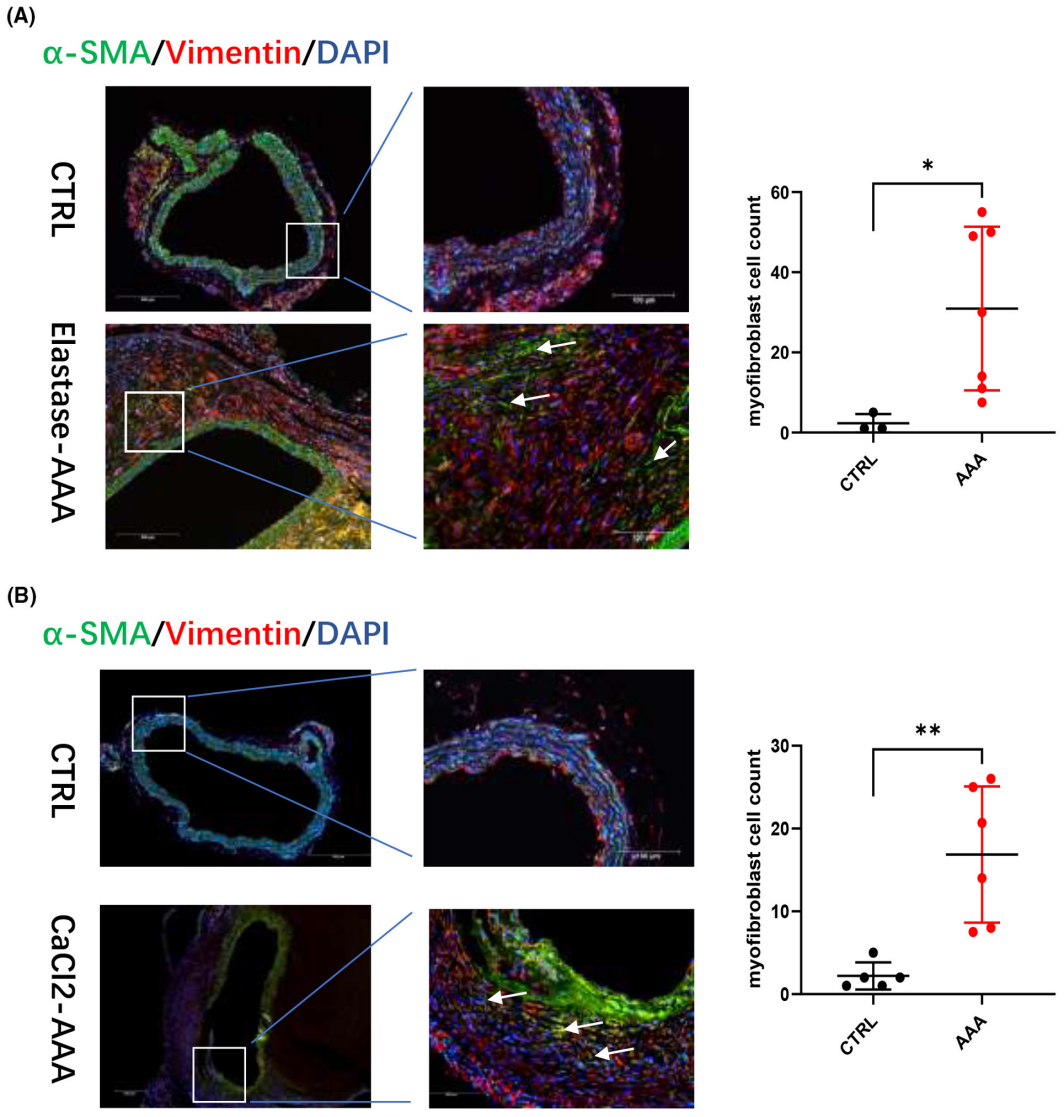
AF phenotypic transformation involved in AAA process. Representative images of α‐SMA and Vimentin immunofluorescence in (A) control aortas (*n* = 3) and elastase‐induced aneurysmal lesions (*n* = 7) and (B) control aortas (*n* = 5) and CaCl_2_‐induced aneurysmal lesions (*n* = 6) green/red/blue colour represented α‐SMA/Vimentin/DAPI respectively;α‐SMA (+) Vimentin (+) was used as a marker of AF transforming into myofibroblasts, myofibroblasts numbers were counted and analysed with Student *t‐*test, scale bars represent 200 μm (left images) and 100 μm (right images), white arrows indicate myofibroblasts location. Values were represented as mean ± SEM; *p < 0.05, **p < 0.01, respectively.

Studies have suggested that phenotypic transformation of AF to myofibroblasts is a significant feature in the pathological process of AAA, which we also confirmed by immunofluorescence staining in elastase‐induced and CaCl_2_‐induced AAA (Figure 3A,B). α‐SMA (−) Vimentin (+) was used as a marker of AF, and α‐SMA (+) Vimentin (+) was used as a marker of AF phenotypic transformation (transformation into myofibroblasts).

### 3.4 YAP1 inhibitor—Verteporfin decreased elastase‐induced and CaCl_2_ ‐induced AAA formation

**FIGURE 4:**
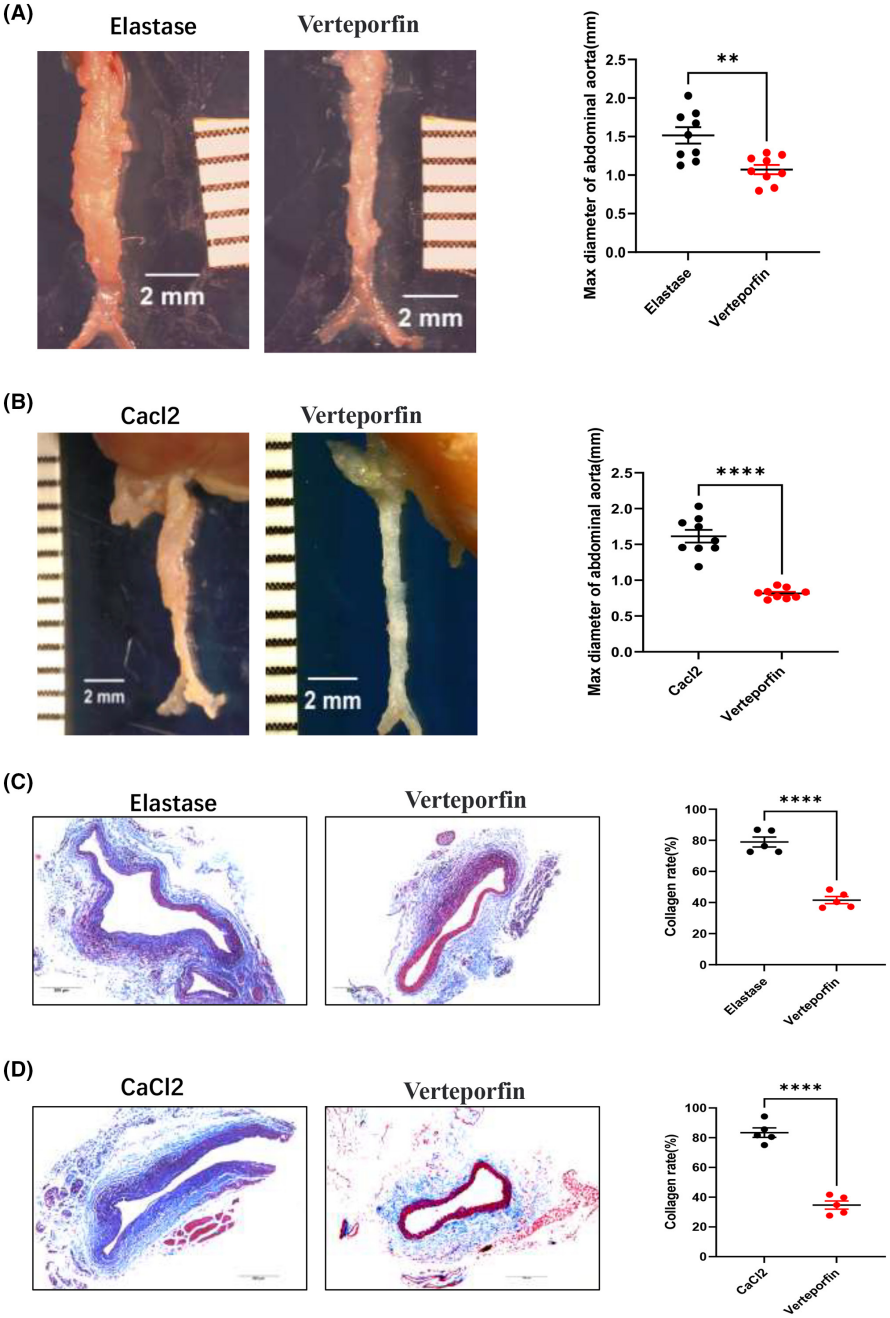
YAP1 inhibitor—Verteporfin decreased elastase‐induced and CaCl_2_‐induced AAA formation and collagen deposition. (A) Representative images of infrarenal abdominal aortas of elastase‐induced AAA (elastase group, *n* = 9) and elastase‐induced AAA model pretreated with Verteporfin (Verteporfin group, *n* = 9); (B) Representative images of infrarenal abdominal aortas of CaCl_2_‐induced AAA (CaCl_2_ group, *n* = 9) and CaCl_2_‐induced AAA model pretreated with Verteporfin (Verteporfin group, *n* = 9). A grid on the scale represents 1 mm, scale bars represent 2 mm. The ex vivo maximum infrarenal abdominal aorta diameter (external diameter) was measured by image J software, maximum diameter difference between Day 14 and baseline in elastase group, CaCl_2_ group and Verteporfin group were calculated and analysed with Student *t‐*test, respectively; (C) Representative images of collagen deposition in infrarenal abdominal aortas of elastase group (*n* = 5) and Verteporfin group (*n* = 5) and (D) Representative images of collagen deposition in infrarenal abdominal aortas of CaCl_2_ group (*n* = 5) and Verteporfin group (*n* = 5) demonstrated by Masson trichrome stain (blue present collagen), scale bars represent 200 μm, the collagen rate was calculated by image J software and analysed with Student *t‐*test. Values were represented as mean ± SEM; **p < 0.01; ****p < 0.0001, respectively.

In order to determine whether YAP1 participates in the formation of AAA, we applied YAP1 inhibitor‐Verteporfin to block the function of YAP1 in the elastase‐induced and CaCl_2_‐induced AAA model, and observed the abdominal aortic dilation and aneurysm formation rate. Mice were randomly divided into four groups: Control group1 (corn oil + elastase treatment, *n* = 9) and Verteporfin (Verteporfin working solution dissolved furtherly in corn oil) group 1 (Verteporfin + elastase treatment, *n* = 9); Control group 2 (corn oil + CaCl_2_ treatment, *n* = 9) and Verteporfin group 2 (Verteporfin + CaCl_2_ treatment, *n* = 9); mice in the Verteporfin group were given Verteporfin (30μg/mouse) by intraperitoneal injection starting from the 24 h before operation every other day until Day 14. We found that in both elastase‐induced and CaCl_2_‐induced AAA model, compared with the control group, the abdominal aorta dilatation was significantly reduced in the Verteporfin group (elastase model: 1.515 ± 0.106 mm vs. 1.072 ± 0.060 mm, *p* < 0.01, Figure 4A); (CaCl_2_ model 1.615 ± 0.088 mm vs. 0.815 ± 0.024 mm, *p* < 0.001, Figure 4B), and the aneurysm formation rate were significantly lower in the Verteporfin groups [elastase model: 22.2% (2/9); CaCl_2_ model 0% (0/9)] than control group [elastase model: 77.8% (7/9); CaCl_2_ model 88.9% (8/9)], *p* < 0.05 in both models.

### 3.5 Verteporfin attenuated collagen deposition in AAA adventitia

Collagen deposition is a major pathological feature of AAA, to assess the effect of Verteporfin on collagen deposition, Masson trichrome stain experiment was performed to compare the collagen rate in abdominal aortas from mice AAA model and Verteporfin treated mice. It is showed that in the elastase‐induced AAA model, collagen rate is much lower in Verteporfin groups (41.6% ± 2.3% vs. 78.9% ± 3.2%, *p* < 0.001, Figure 4C); and in the CaCl_2_‐induced AAA model, collagen rate is also decreased in Verteporfin groups (34.7% ± 2.7% vs. 83.4% ± 3.2%, *p* < 0.001, Figure 4D).

### 3.6 Verteporfin inhibited AF phenotype transformation and migration

**FIGURE 5:**
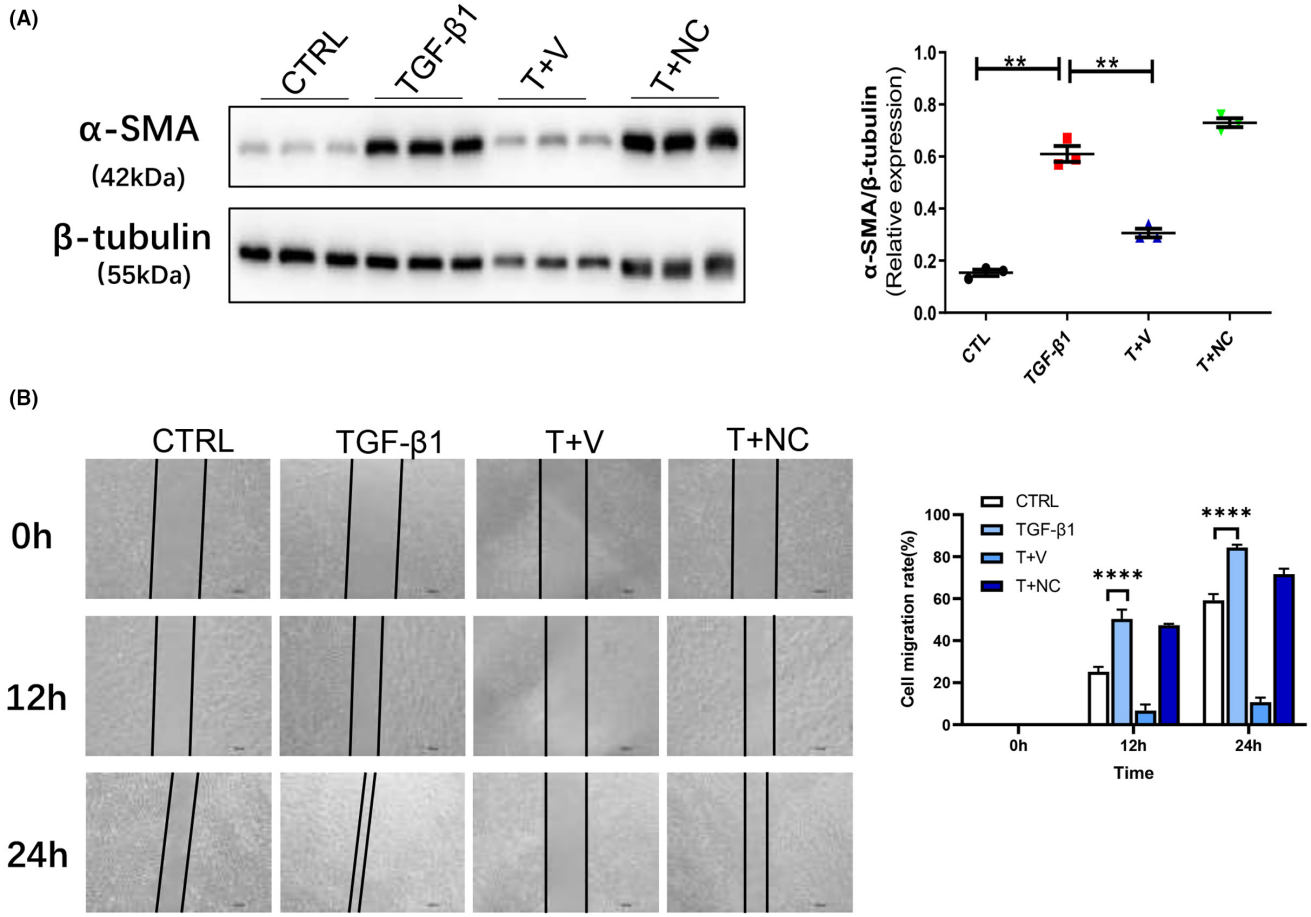
Verteporfin inhibited AF phenotype transformation and migration. (A) Protein abundance of α‐SMA in AF incubated with TGF‐β1 (10 ng/mL) and pretreated 24 h with Verteporfin (1 μM, dissolved with 0.1%DMSO) or negative control (NC, 0.1% DMSO). (B) Representative images of migration distance of AF incubated with TGF‐β1 (10 ng/mL) and pretreated 24 h with Verteporfin (1 μM, dissolved with 0.1% DMSO) or negative control (NC, 0.1% DMSO), respectively. Scale bars represent 200 μm. Values were represented as mean ± SEM; One Way ANOVA was used for data analysis in (A, B), **p < 0.01; ****p < 0.0001, respectively. T represent TGF‐β1, V represent Verteporfin, NC represent negative control (0.1% DMSO in A, B).

The AF was pretreated with Verteporfin (1 μM) for 24 h, followed by incubated with TGF‐β (10 ng/mL) for 24 h; we found that AF transformation and migration were significantly inhibited by Verteporfin (Figure 5A,B).

## 4 DISCUSSION

**FIGURE 6:**
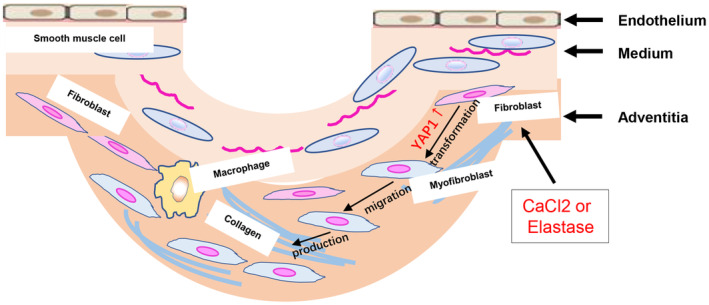
The role of YAP1 in AAA development. This graphic demonstrated the mechanism of YAP1 in AAA development: In both elastase and CaCl_2_‐induced AAA models, these stimulus upregulated YAP1 expression, then YAP1 initiated the process of AF transforming into myofibroblasts and migration, YAP1 might also enhanced the process of collagen deposition in aortas, all these together promoted AAA formation.

In this study, we used elastase‐induced and CaCl_2_‐induced AAA model demonstrated that YAP1 promoted the development of AAA, and blocking YAP1 in vivo by YAP1 inhibitor can significantly reduce the incidence of AAA and collagen deposition. In both AAA models, YAP1 were upregulated in abdominal aortas; in vitro study, we confirmed that YAP1 initiated the process of AF transforming into myofibroblasts and migration, YAP1 also enhanced the process of collagen deposition in aortas, all these together promoted AAA formation (Figure 6).

At present, studies on the adventitia of AAA have mainly focused on inflammatory cell infiltration, there are a few studies on AF, the role of AF in AAA has not been clarified. The adventitia of the vessel was considered as a scaffold until recently, when studies confirmed that the adventitial plays a complex and essential role in various vascular diseases. As the most abundant cell in adventitia, AF is the first sensor to environmental stimuli around vascular. Once activated, multiple response such as proliferation, secretion of cytokines and growth factors, alteration of extracellular matrix and expansion of vasa vasorum can occur.^13^ In addition, AF has significant plasticity, can be transformed into myofibroblasts, participate in vascular remodelling, secrete inflammatory factors, transform into pro‐inflammatory type and recruit monocytes and macrophages,^14^ which also involved in the pathologic process of AAA.^5^ Hyperhomocysteinemia (HHcy) promoted Ang II‐induced AAA information, it is showed that proinflammatory IL‐6 and MCP‐1 were colocalized with AF in HHcy and Ang II mice, Hcy sequentially stimulated AF transformation into myofibroblasts, confirmed the essential role of AF in AAA; cylindromatosis (CYLD) also aggravated CaPO_4_‐induced AAA by inducing AF activation (secret proinflammatory cytokines) and transformation.^11^ Consistent with previous studies, we found AF transformation contributed to AAA development, and YAP1 activated AF phenotype transformation and migration, which were remarkably blocked by YAP1 inhibition through YAP1 inhibitor‐Verteporfin; more critically, intraperitoneal injection of Verteporfin significantly attenuated collagen deposition, alleviated AAA development, reduced AAA incidence, Verteporfin might be a very promising clinical drug for AAA therapy.

YAP1 and TAZ are mechanosensitive transcriptional coactivators, respond to substrate stiffness, cell density and shear stress. YAP1 contributed to various of vascular diseases, such as atherosclerosis,^15^, ^16^, ^17^, ^18^ hypertension,^19^ vascular injury, angiogenesis and aneurysm.^20^, ^21^, ^22^, ^23^, ^24^ Studies on the mechanism of YAP1 involved in vascular diseases mainly focus on the endothelial cell (EC) and the vascular smooth muscle cell (VSMC), YAP1 contributed to EC dysfunction in the process of atherosclerosis, EC proliferation in hypertension and angiogenesis; YAP1 also induced VSMC proliferation, migration and phenotype transformation in vascular diseases.^25^ The role of YAP1 in aneurysm has not been elucidated clearly. It is suggested that YAP1 expression reduced in patients with ascending aortic aneurysms and VSMCs, which was associated with VSMCs apoptosis and extracellular matrix (ECM) disorders of the media.^24^ In this study, we confirmed that YAP1 expression upregulated in human AAA tissues through both western blotting and immunohistochemistry, furtherly, we found YAP1 mainly increased in AF in AAA, so we concentrated upon the influence of YAP1 on AF in following research.

The effect of YAP1 on AF has not been fully illustrated. YAP1 has been studied in pulmonary hypertension; it is suggested that YAP/TAZ activation may play a role in initiating pulmonary vascular ECM remodelling by activating pulmonary adventitial fibroblast proliferation in response to increased pulsatility and shear stress.^8^ YAP1 promoted AF proliferation and impaired AF apoptosis in pulmonary arteries,^26^ controlled ECM remodelling, consequently initiated pulmonary hypertension.^27^ Although the current number of studies is small, it is worth believing that AF also play a key role in AAA.^5^, ^11^, ^28^, ^29^ Indeed, our study confirmed that AF transformation and migration involved in AAA lesions, which may be facilitated by YAP1; in vivo study showed that YAP1 inhibitor‐Verteporfin diminished AF transformation to myofibroblast and migration; in vitro study proved furtherly that YAP1 inhibitor‐verteporfin remarkably attenuated the process of AF transformed to myofibroblast by inhibiting α‐SMA expression and migration demonstrated by wound healing experiment.

Above all, this study exhibited for the first time that YAP1 involved in AAA development as far as we known by regulating AF function, it is trustworthy that YAP1 could be promising drug therapy target for AAA patients. Meanwhile, the specific mechanism of YAP1 influenced AF function should be further in‐depth studied.

## AUTHOR CONTRIBUTIONS

**Cuiping Xie:** Conceptualization (lead); data curation (lead); formal analysis (lead); funding acquisition (lead); investigation (lead); methodology (lead); project administration (lead); resources (lead); software (lead); supervision (lead); validation (lead); visualization (lead); writing – original draft (lead); writing – review and editing (lead). **Yanting Hu:** Data curation (equal); formal analysis (equal); investigation (equal); methodology (equal); writing – original draft (supporting); writing – review and editing (equal). **Zhehui Yin:** Investigation (equal); methodology (equal); project administration (equal).

## CONFLICT OF INTEREST STATEMENT

The authors declare they have no conflict of interest.

## Data Availability

All data are contained within the manuscript.
